# Use of continuous infusion technique with pre-filled elastic pumps for prevention of centrally inserted central catheter occlusion in critically ill patients: A feasibility study

**DOI:** 10.1097/MD.0000000000040930

**Published:** 2024-12-13

**Authors:** Wei Jia, Kaiping Wu, Kaifei Song, Wenjuan Yun, Jin Wang, Yaping Yi, Lingyun Xu

**Affiliations:** a Department of Breast Surgery, The Affiliated Changzhou Second People’s Hospital of Nanjing Medical University, Changzhou, China; b Changzhou Medical Center, Nanjing Medical University, Changzhou, China; c Intensive Care Unit, The Affiliated Changzhou Second People’s Hospital of Nanjing Medical University, Changzhou, China; d Department of Nursing, The Affiliated Changzhou Second People’s Hospital of Nanjing Medical University, Changzhou, China.

**Keywords:** catheter obstruction, central venous catheters, continuous infusion, intensive care units, pulsatile flow

## Abstract

**Background:**

Flushing catheter seems to be a crucial procedure for preventing centrally inserted central catheter (CICC) occlusion, which can flush the liquid and blood attached to the lumen into the bloodstream. The continuous infusion technique is characterized by continuous perfusion of flushing fluid and maintaining positive pressure in the lumen, which has been demonstrated to effectively prevent peripheral indwelling needle occlusion. However, the use of continuous infusion technique in CICC care among critically ill patients has been rarely described.

**Objective:**

To determine the feasibility and direct outcomes of continuous infusion technique in preventing CICC occlusion among critically ill patients.

**Methods:**

Participants from the intensive care unit who had a new centrally inserted central catheter placed within 24 hours were randomly assigned to 2 groups at a ratio of 1:1. They received pulsed infusion or continuous catheter infusion using pre-filled elastic pumps. During the trial period, on days 3 and 7 and whenever there were signs of catheter occlusion, whether the catheter occlusion was evaluated by the Catheter Injection and Aspiration Classification. Moreover, nurses meticulously observed the incidence of catheter-related complications, altered vital signs, and dysfunctions of elastic pump.

**Results:**

The catheter occlusion rates were 17.4% and 43.5% in participants who received continuous infusion (n = 23) and those who received pulsed infusion (n = 23), respectively. There was no significant difference in catheter occlusion rate between the 2 groups (*χ*² = 3.696, *P* = .06). The Kaplan–Meier curve results showed that the cumulative probability of central catheter occlusion events (within 7 days) in the continuous infusion group decreased (*χ*²=4.322, Log rank *P* = .04). Moreover, multivariate Cox regression indicated that the continuous infusion can reduce the risk of occlusion within 7 days by 91.8% (hazard ratio = 0.082, 95% confidence interval [0.014–0.487], *P* = .006). During the trial, no cases of detrimental altered vital signs and catheter-related complications in addition to occlusion were documented.

**Conclusions:**

In this study, continuous infusion technique with pre-filled elastic pumps was successfully used in critically ill patients for prevention of CICC occlusion, without major undesired effects. A larger cohort and a randomized clinical trial are warranted in order to establish its absolute efficacy in CICC care.

## 1. Introduction

In the intensive care unit (ICU), approximately 50% of patients require a central venous access device for hemodynamic monitoring, blood transfusion or massive rapid rehydration.^[1]^ Centrally inserted central catheters (CICCs) have emerged as the preferred choice for in-hospital use at most facilities, mainly because of its low cost.^[2]^ However, critically ill patients are at risk for catheter occlusion due to factors related to their abnormal coagulation systems and their complex medical needs.

The specific manifestations of catheter occlusion include the formation of fibrin sheaths, blood clots, or drug deposits on the lumen surface, which ultimately leads to a decline in the patency of catheter infusion. Among them, the inability to administer drugs from the lumen and withdraw blood is regarded as complete occlusion, while the situation in which drugs can still be administered from the lumen but blood withdrawal fails is considered partial occlusion. The occurrence of catheter occlusion is closely associated with multiple factors, such as the type and indwelling duration of catheter, the infusion of drugs, and the patient’s disease status.^[3]^ Studies have reported the incidence of CICC-related occlusion ranging from 14% to 36%.^[4]^ Catheter occlusion not only impairs the continuity of treatment for critically ill patients, but also increases the risk of catheter-related bloodstream infection (CRBSI) and thromboembolism, resulting in additional thrombolytic therapy or catheter replacement, augmenting patient discomfort and medical costs.^[5]^ The prevention of CICC-related occlusion requires a multifaceted approach that includes the enhancement of patient-related cognition, appropriate catheter insertion and catheter flushing technique.^[6]^

Flushing catheter seems to be an important step during catheter care, which maintains catheter patency by removing deposits from the endoluminal wall of the catheter.^[7]^ Previous studies have shown that hydrodynamics has a determinant effect on the cleaning efficacy.^[8]^ The pulsed infusion technique, in which saline is injected by repeatedly pressing and stopping several times, generates turbulence in the catheter for a more powerful cleaning effect.^[9]^ In recent years, the pulsed infusion technique has gained widespread adoption, which is also recommended by the Infusion Therapy Standards of Practice.^[10]^ However, it is not fully suitable or desirable for critically ill patients with a high risk of occlusion, especially those who need continuous and accurate infusion of sedation or vasoactive drugs. This phenomenon underlines the imperative of enriching catheter flushing techniques.

The continuous infusion technique, in which saline is continuously infused into the lumen at a constant flow rate, maintains the pressure in the lumen higher than the venous pressure, in order to reduce blood reflux and the adhesion of infused fluid deposits, fibrin molecules.^[11]^ Previous studies have applied the continuous infusion technique to peripheral venous catheters, showing comparable or even superior efficacy in preventing catheter occlusion when compared with pulsed infusion.^[12,13]^ However, the use of continuous infusion technique in centrally inserted central catheter care among critically ill patients has been rarely described. This study employed a pre-filled elastic pump based on the continuous infusion technique to continuously and slowly infuse the flushing fluid into the CICC at an accurate rate (2 mL/h). The aim was to determine the feasibility and direct outcomes of prophylactic continuous low-speed flushing CICC in critically ill patients.

## 2. Methods

### 2.1. Study design

The study is a feasibility and randomized controlled clinical trial, which was conducted in the Intensive Care Unit of The Affiliated Changzhou Second People’s Hospital of Nanjing Medical University from March 2023 to November 2023. The study was granted approval by the Hospital Ethics Review Board (No.: YLJSA052). Before the study procedures, all participants or their close relatives provided written informed consent. The procedures followed in this study were in line with the principles of the “Helsinki Declaration.”

### 2.2. Study participants

This study included patients aged 14 to 80 years admitted to ICUs who had a new centrally inserted central catheter placed within 24 hours. The central catheters of participants had to be placed via internal jugular vein or subclavian vein, and catheter tips were confirmed by X-ray to be located in the superior vena cava. Moreover, the catheters were in a normal functional state (injection ability 1, aspiration ability 1 as judged by Catheter Injection and Aspiration Classification), and were expected to be used for more than 8 days.^[14]^ The exclusion criteria were: patients who must use alternative fluids for catheter locking; patients who need to use drugs that are incompatible with normal saline; patients who need to use drugs that cannot tolerate the cessation of infusion for catheter flushing. The elimination criteria were: patients who were removed from the ICU during the trial period; patients who had a device removal; patients who withdrew from this study (including death); patients who did not receive the appropriate participant interventions after randomization.

### 2.3. Randomization

Participants were randomly assigned to the continuous infusion group and the pulsed infusion group. Randomization method: The data manager responsible for randomization generated a 1:1 random sequence and the corresponding grouping information according to the sample size. These random numbers were concealed in numbered opaque envelopes. When confirming the participants’ inclusion in this study, the researchers would open the envelopes in order to obtain the corresponding random numbers and grouping outcomes.

### 2.4. Intervention

**Figure 1.**
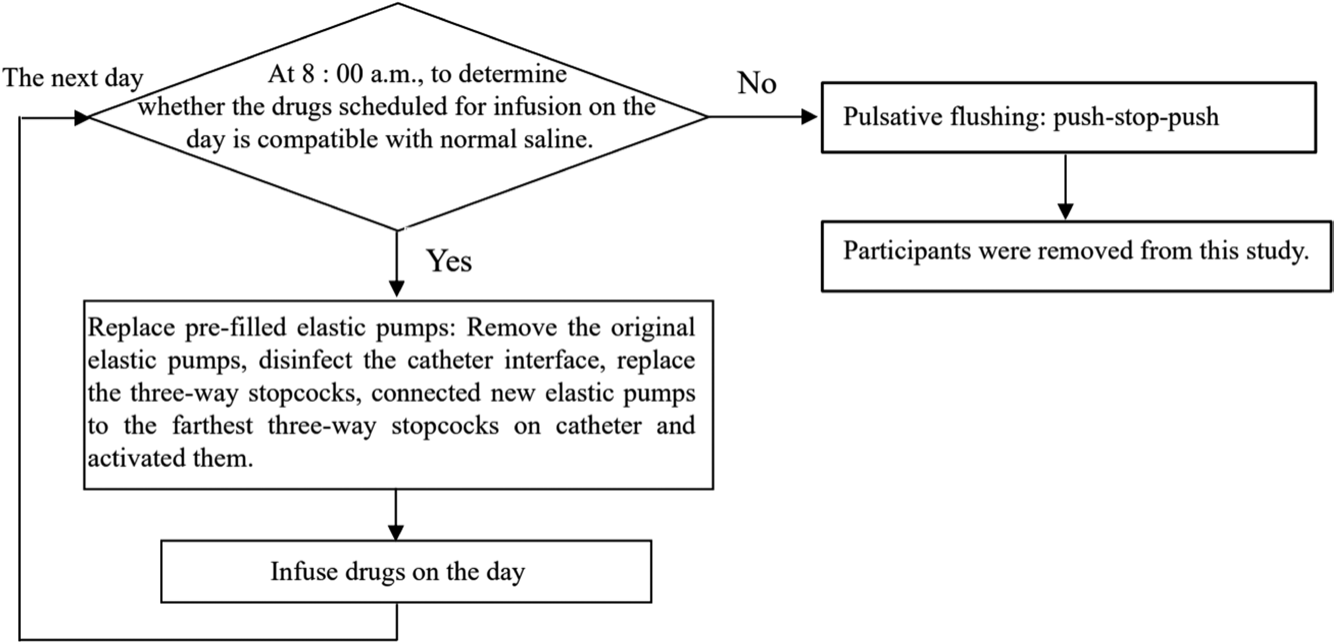
The operation flow chart of catheter flushing in the continuous infusion group.

The continuous infusion group: The low-speed continuous infusion using the pre-filled elastic pump (Jiangsu Jinta Pharmaceutical Company, Jiangsu, China) was carried out. Every morning at 8 o’clock, trained ICU nurses connected the elastic pumps to the farthest three-way stopcocks on both sides of the CICC lumen and activated them (the detailed operation process is shown in Fig. 1). The elastic pump was pre-filled with 50 mL normal saline as the flushing fluid, and a flow restrictor was present inside. After activation, it would continuously infuse the flushing fluid into the lumen at a constant rate of 2 mL/h, thereby establishing a closed perfusion system connected to the centrally inserted central catheter to maintain the infusion pressure within the lumen. At 8 o’clock the following morning, the elastic pump was replaced in accordance with the same process. The device is small in size and will not impede the early rehabilitation exercises of critically ill patients.

**Figure 2.**
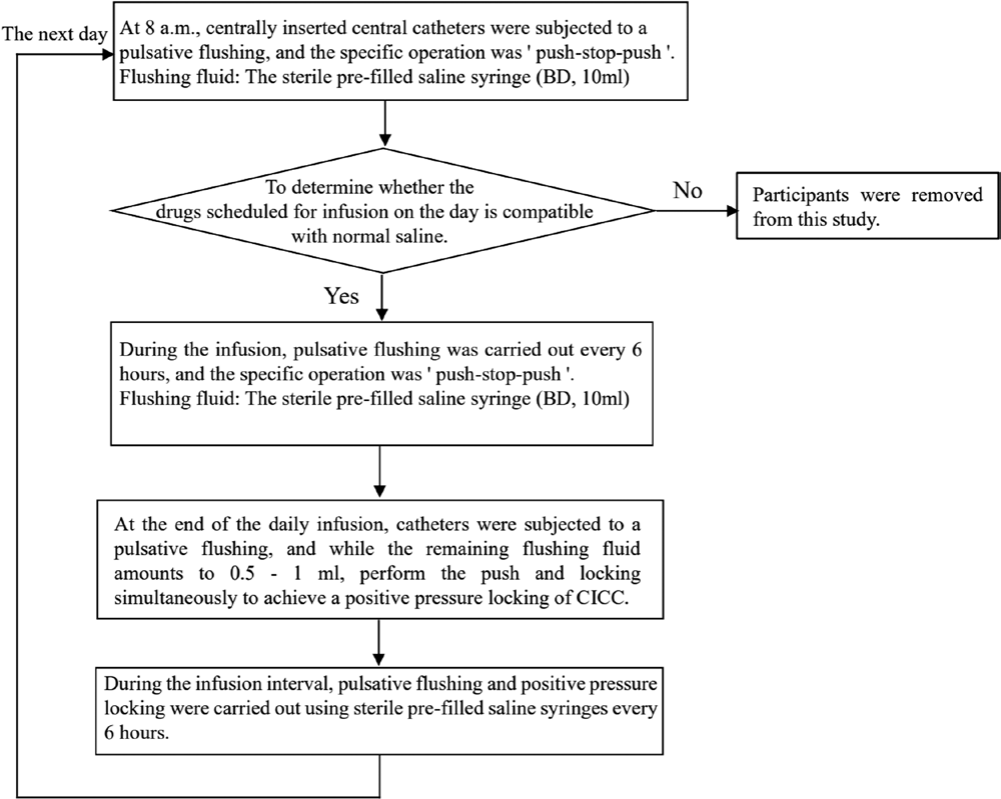
The operation flow chart of catheter flushing in the pulsed infusion group.

The pulsed infusion group: The manually pulsed infusion using the sterile pre-filled saline syringe was carried out. Every morning at 8 o’clock, ICU nurses manually performed pulsative flushing on centrally inserted central catheter and started the daily infusion. During the infusion, pulsative flushing was carried out every 6 hours. At the end of the daily infusion, the catheter was flushed and subsequently locked with positive pressure. During the infusion interval, the flushing and positive pressure locking were conducted every 6 hours (the detailed operation process is shown in Fig. 2). The nurses were required to operate the bilateral lumens at the same time with one hand, to prevent the return of blood in other lumens caused by washing only one lumen.

In order to ensure the reliability and accuracy of the research results, before implementation, homogenization training was carried out for ICU nurses who participated in the study, including standardized operation processes for catheter flushing and locking, connection and replacement processes of elastic pumps, alarm identification of elastic pumps, etc. Ensure that each nurse masters the operation process and precautions. Additionally, all centrally inserted central catheters were inserted by experienced intensive care doctors in accordance with the standardized catheterization process under ultrasound guidance, and catheter tips were confirmed by X-ray to be located in the proximal third of the superior vena cava. All participants’ indwelling catheters were double-lumen disposable central venous catheters (Zhuhai Funia Medical Equipment Co., Ltd., Zhuhai, China). Catheter model: CVC-27F 20, length 20 cm, diameter 2.4 mm. The infusion connectors were all of the same type of positive pressure, needle-free infusion connectors (BD Luer-Lok™ 394605, Gurgaon, Haryana, India) with the split septum as the internal mechanism.

### 2.5. Outcome measures

In this study, the Catheter Injection and Aspiration (CINAS) classification was used to evaluate centrally inserted central catheter occlusion based on 2 dimensions: injection and aspiration.^[14]^ Each dimension is divided into 3 grades according to the degree: Grade 1, easy; Grade 2, difficult; Grade 3, impossible. “Easy” indicates that the catheter is very unobstructed, and there is no resistance to inject or aspirate. ``Difficulties’’ indicates that there is resistance to injection, or intermittent blood return during aspiration. “Impossible” indicates that either injection is unfeasible or blood aspiration is unachievable. “X” is the fourth grade, indicating that it cannot be evaluated. The CINAS results can form 16 combinations (see Fig. S1, Supplemental Digital Content, http://links.lww.com/MD/O174). IN1AS1 represents good catheter function, and other results represent different catheter dysfunction, which is also described as occlusion. On days 3 and 7 of the trial period, or when there were signs of occlusion, such as liquid gravity drop speed < 60 drops/min or electronic infusion pump occlusion alarm, nurses would use CINAS to evaluate the catheter. If catheter occlusion is confirmed, the researchers follow the “Hospital Centrally Inserted Central Catheter Occlusion Management Guidelines” for processing. If the catheter is still unable to restore patency after processing, the catheter would be considered for removal and recorded in the case report.

As part of clinical care, nurses meticulously observe the incidence of complications related to occlusion, including CRBSIs, catheter-related thrombosis, and accidental device removal. When patients present with clinical manifestations of sepsis, such as high fever, blood samples are collected from both the catheter and peripheral veins for paired blood cultures to determine whether CRBSIs have occurred. When patients present with pain, edema, erythema, and other symptoms associated with venous blood flow obstruction, the advanced practice registered nurses responsible for intravenous treatment would use color flow Doppler ultrasound to confirm whether catheter-related thrombosis has occurred. In addition, accidental device removal is defined as the catheter remaining in place for less than the expected duration and being accidentally removed before the end of treatment.

Safety and feasibility were assessed through the following aspects: (1) altered heart rate and/or blood pressure caused by continuous infusion, (2) the dysfunction of elastic pump, including leakage of flushing fluid, too fast or too slow flushing speed, etc. In the event of the above incidents, nurses requested another researcher for secondary confirmation and timely processing, and the cases were recorded in the case report.

### 2.6. Statistical analysis

The Shapiro–Wilk test was used to test whether the continuous variables conformed to a normal distribution. Variables with normal distribution were expressed as mean ± standard deviation, and independent samples *t* test was used for comparison among groups. Variables with non-normal distribution were expressed as median and interquartile range, and nonparametric Mann–Whitney *U* test was used for comparison among groups. The categorical variables were expressed as frequency and percentage, and Chi-square test or Fisher exact test was used for comparison among groups. Taking catheter occlusion as the endpoint event, the Kaplan–Meier method was used to draw the survival curve to describe the occurrence of occlusion in the 2 groups. Herein, the Log-Rank test was used to compare the difference between the groups. Subsequently, the Cox regression model was used to assess the risk of occlusion in both groups, and the results were expressed as a hazard ratio with 95% confidence interval. The Cox regression model included possible influencing factors of occlusion, such as age, gender, body mass index, etc. All statistical analyses were performed using SPSS Software version 24 (IBM^®^). Two-sided *P* < .05 was considered statistically significant. Additionally, the types and incidence of elastic pump failure incidents during the trial period were described.

## 3. Results

**Figure 3.**
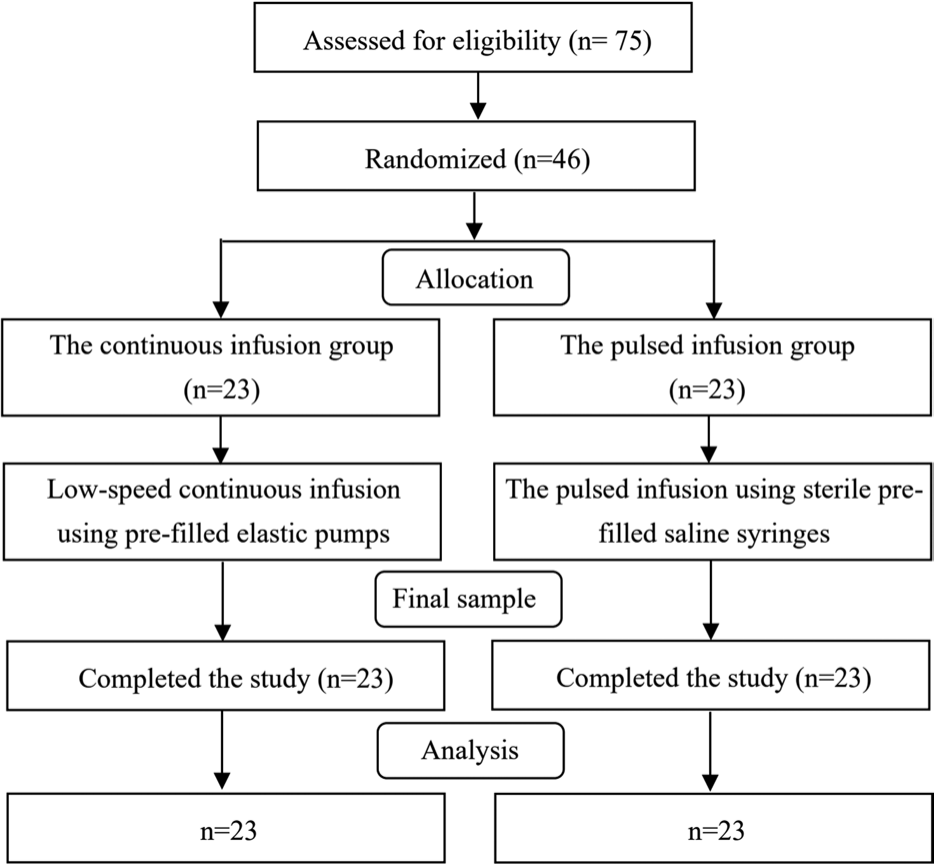
Study flow chart.

Forty-six participants were randomly assigned to either the continuous infusion group or the pulsed infusion group, with 23 in each group, and all of them completed the study. The study flow chart is shown in Figure 3. The median age of participants was 67.05 years old, and the median body mass index was 24.28 kg/m². The majority of participants (91.3%) had centrally inserted central catheters inserted through the internal jugular vein. All baseline demographic/disease characteristics/special drug infusions were balanced between study arms (Table 1).

**Table 1:** Baseline characteristics of participants.

	The continuous infusion group	The pulsed infusion group	*P*
	(n = 23)	(n = 23)	
Age, years, median (Q1, Q3)	67.85 (59.41, 72.87)	66.25 (52.81, 77.01)	.65*
Male, n (%)	12 (52.2%)	7 (30.4%)	.13†
BMI, kg/m^2^, median (Q1, Q3)	23.31 (20.31, 25.14)	24.68 (21.20, 27.31)	.07*
Main reason for ICU admission, n (%)			
Pneumonia	4 (17.4%)	6 (26.1%)	.38‡
Abdominal pain	4 (17.4%)	2 (8.7%)	
Acidosis	3 (13.0%)	2 (8.7%)	
Pancreatitis/cholecystitis	5 (21.7%)	4 (17.4%)	
Digestive tract perforation	1 (4.4%)	2 (8.7%)	
Multiple site damage	0 (0%)	4 (17.4%)	
Other reasons	6 (26.1%)	3 (13.0%)	
Catheter insertion location, n (%)			
Subclavian vein	2 (8.7%)	2 (8.7%)	–
Internal jugular vein	21 (91.3%)	21 (91.3%)	
APTT, seconds, median (Q1, Q3)	29.10 (25.90, 32.50)	28.70 (26.10, 31.60)	.99*
PT, seconds, median (Q1, Q3)	12.80 (12.30, 13.90)	13.50 (11.80, 13.90)	.58*
INR, median (Q1, Q3)	1.14 (1.08, 1.22)	1.17 (1.05, 1.24)	.71*
FIB, g/L, median (Q1, Q3)	4.23 (2.95, 5.24)	4.51 (2.95, 6.99)	.61*
Platelet count, median (Q1, Q3)	273.00 (177.00, 291.00)	194.00 (118.00, 309.00)	.50*
Mechanical ventilation, n (%)			
Yes	10 (43.5%)	12 (52.2%)	.56†
No	13 (56.5%)	11 (47.8%)	
Parenteral nutrition infusion, n (%)			
Yes	5 (21.7%)	4 (17.4%)	.71†
No	18 (78.3%)	19 (82.6%)	

APTT = activated partial thromboplastin time, BMI = body mass index, FIB = fibrinogen, ICU = intensive care unit, INR = international normalized ratio, PT = prothrombin time.

* Wilcoxon Mann–Whitney test.

† Chi-square test.

‡ Fisher exact test.

**Figure 4.**
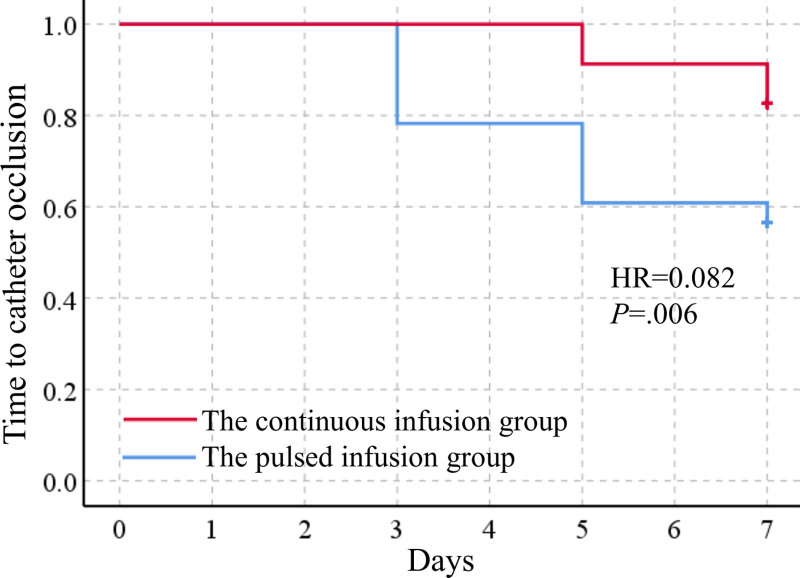
Kaplan–Meier “survival” curve for time to catheter occlusion through 7 days.

Of the participants in the continuous infusion group, 17.4% (n = 4) had centrally inserted central catheter occlusion within 7 days, while this rate was 43.5% (n = 10) in the pulsed infusion group. There was no significant difference in catheter occlusion rate between the 2 groups (*χ*²=3.696, *P* = .06). All the occluded catheters recovered after the process and continued to be used. The Kaplan–Meier ``survival’’ curve showed that, compared with the pulsed infusion group, the cumulative probability of centrally inserted central catheter occlusion events (within 7 days) in the continuous infusion group decreased (*χ*²=4.322, Log rank *P* = .04) (Fig. 4). Moreover, multivariate Cox regression indicated that the continuous infusion using elastic pumps and the parenteral nutrition infusion were independent influencing factors of catheter occlusion (Table 2). Specifically, the continuous infusion can reduce the risk of occlusion within 7 days by 91.8% (hazard ratio = 0.082, 95% confidence interval [0.014–0.487], *P* = .006).

**Table 2:** COX regression analysis of centrally inserted central catheter occlusion.

Parameters	Wald *χ*^2^	HR	95% CI	*P*
Continuous infusion	7.571	0.082	0.014–0.487	.006
Male	0.156	1.472	0.216–10.026	.69
Age	0.016	0.995	0.914–1.083	.90
BMI	1.502	1.200	0.897–1.605	.22
PT	0.781	0.783	0.456–1.346	.38
APTT	0.077	1.032	0.825–1.292	.78
FIB	0.266	0.875	0.526–1.455	.61
Platelet count	0.591	1.004	0.994–1.014	.44
Parenteral nutrition	6.434	10.465	1.705–64.216	.01
Mechanical ventilation	0.001	1.022	0.225–4.638	.98

95% CI = 95% confidence interval, APTT = activated partial thromboplastin time, BMI = body mass index, FIB = fibrinogen, HR = hazard ratio, PT = prothrombin time.

During the trial, none of the participants experienced CRBSIs, catheter-related thrombosis, or accidental device removal. No cases of altered heart rate and/or blood pressure were documented, and continuous infusion was well tolerated in all participants. One of the 23 participants who used the elastic pump for continuous infusion had leakage of the flushing fluid. This failure was proven to be caused by the loose connection and was not related to the operational status of the elastic pump. Due to timely discovery and resolution, this incident did not adversely affect the participants.

## 4. Discussion

Data from this study appear to support the feasibility and efficacy of low-speed continuous infusion using elastic pumps to prevent centrally inserted central catheter occlusion in critically ill patients. This study demonstrated that no statistically significant difference was observed in the catheter occlusion rate within 7 days between the continuous infusion group and the pulsed infusion group. In addition, the Kaplan–Meier “survival” curve showed that patients receiving continuous infusion had a significantly lower cumulative risk probability of catheter occlusion during the trial period, and their catheter indwelling time before occlusion was significantly longer. And the Cox regression model also showed that continuous infusion using elastic pumps could significantly reduce the risk of catheter occlusion. This finding is consistent with the results of a multicenter randomized controlled trial involving 250 patients who inserted central venous catheters.^[11]^ Another study on PICC and midline catheters also showed that the utilization of an elastomeric continuous infusion device could reduce the incidence of catheter occlusion by 50% compared with the traditional flushing device.^[15]^

Due to the particularity of disease and treatment, critically ill patients usually have multiple risk factors leading to CICC occlusion. In terms of catheter selection, double-lumen or three-lumen centrally inserted central catheters are usually preferred to address the complex treatment requirements, and they have a higher occlusion probability compared to single-lumen catheters.^[16]^ Secondly, long-term bed rest, reduced limb activity, and relaxation of skeletal muscle in critically ill patients will also lead to venous blood stasis, which will promote the occurrence of CICC-related occlusion. If patients are complicated with hypertension, diabetes, and infection, the occlusion risk will further increase.^[17]^ In addition, as found in this study, parenteral nutrition infusion is a risk factor for CICC occlusion. It may be because the infusion of high concentration nutrient solution will increase the blood viscosity and slow down the blood flow velocity. Furthermore, lipid emulsion is the most commonly used parenteral nutrition preparation, and lipochondria will accumulate due to changes in electrolyte and pH levels, ultimately resulting in catheter occlusion.^[18]^

The existence of high occlusion risk puts forward higher requirements for catheter care, especially catheter flushing. Continuous infusion technique has been controversial in the past, and there is limited evidence supporting its application in CICCs. An in vitro experiment used a mixture of fibronectin and bovine to simulate the physiological protein deposition in peripheral venous catheters. It was found that the removal effect of pulsed infusion was better than that of low-speed continuous infusion.^[19]^ But some studies, including this one, have demonstrated that continuous infusion was not inferior to pulsed infusion regarding occlusion prevention. The elastic pump infuses the flushing fluid at a constant low speed in a closed manner, maintaining the centrally inserted central catheter lumen in a positive pressure and open state throughout, thus avoiding blood reflux during infusion and thrombus deposition at the tip of the catheter after locking. Additionally, the flushing solution, as an additional carrier, may accelerate the flow rate of the infusion liquid in the lumen to a certain extent, and reduce the adhesion of sediment in the lumen. Compared with pulsed infusion, the effect achieved by continuous infusion is less dependent on the nurse’s operation specifications. Constant infusion speed in the device enables similar flushing effects to be achieved in different populations. It is worth mentioning that the speed is an important parameter in optimizing flushing efficiency. Currently, continuous infusion speed implemented ranges from 0.5 to 50 mL/h. If the infusion speed is too slow, it may reduce the flushing effect. If the infusion speed is too fast, it may lead to overhydration and even altered blood pressure. In this study, the infusion speed of 2 mL/h did not lead to alterations in vital signs and was safe and feasible for patients. In the future, researchers could design relevant in vitro experiments in accordance with the laws of fluid dynamics and comprehensively consider the length and inner diameter of the lumen to determine the optimal infusion speed.

Although the setting of infusion speed lacks relevant standards, continuous infusion technique is still widely welcomed by nurses. It may be because it saves nursing operation time compared with manual pulsed infusion. A survey of Canadian nurses found that >50% of respondents often use this technique to keep venous catheters open.^[20]^ But in resource-scarce public hospitals, economic factors play a key role when selecting a catheter flushing device, considering the higher cost of pre-filled elastic pumps. Future researchers could compare the cost-effectiveness of different flushing devices in practical clinical settings, based on a comprehensive analysis of device costs, nursing operation time, the rate of adverse events and the cost of treating them.

No catheter-related adverse consequences such as CRBSI and catheter-related thrombosis occurred in this study. Only one participant had flushing fluid leakage. We believe that this incident was caused by the operator’s failure to closely connect the pre-filled elastic pump to centrally inserted central catheter. After this incident, we reemphasized the connection steps of the elastic pump, and no adverse safety incidents occurred subsequently.

This study had some limitations. The sample size was small, the follow-up assessments were short-term. It is necessary to evaluate the effects of continuous infusion on centrally inserted central catheter occlusion in a future randomized controlled trial with a large sample and longer-term follow-ups.

## 5. Conclusions

In conclusion, this preliminary study highlighted that the use of continuous infusion technique in critically ill patients is safe and appears to be a promising approach in the prevention of catheter occlusion. Continuous infusion technique using the pre-filled elastic pump could be used for preventing centrally inserted central catheter occlusion even in this high-risk population, however, further research with a multi-center randomized controlled trial is warranted to prove its absolute efficacy in the prevention of CICC occlusion.

## Author contributions

**Conceptualization:** Wei Jia, Yaping Yi.

**Data curation:** Kaiping Wu, Kaifei Song.

**Formal analysis:** Wei Jia, Kaiping Wu, Lingyun Xu.

**Investigation:** Kaifei Song, Wenjuan Yun, Jin Wang.

**Methodology:** Wei Jia, Kaiping Wu, Wenjuan Yun, Lingyun Xu.

**Project administration:** Kaiping Wu, Kaifei Song.

**Supervision:** Kaiping Wu, Yaping Yi.

**Writing – original draft:** Wei Jia.

**Writing – review & editing:** Wei Jia, Kaiping Wu, Kaifei Song, Lingyun Xu.

## Supplementary Material



## References

[1] DiPietro LM Gaies M Banerjee M. Central venous catheter utilization and complications in the Pediatric Cardiac ICU: a report from the Pediatric Cardiac Critical Care Consortium (PC4). Pediatr Crit Care Med. 2020;21:729–37. 32453921 10.1097/PCC.0000000000002306

[2] de Souza Fantin S Scherer Dos Santos M Ferro EB Hirakata VN Ferreira de Azeredo da Silva A Rabelo-Silva ER. Peripherally inserted central catheter versus centrally inserted central catheter for in-hospital infusion therapy: a cost-effectiveness analysis. Value Health Reg Issues. 2024;41:123–30. 38401289 10.1016/j.vhri.2023.12.006

[3] Taglialatela I Mariani L Dotti KF. Central venous catheters-related-thrombosis and risk factors in oncological patients: a retrospective evaluation of recent risk scores. Tumori. 2023;109:363–9. 35815563 10.1177/03008916221111419 PMC10363937

[4] Irigoyen PM Jimenez MG Arellano EML Pérez MS Cabredo RV. Patency, assessment, and management of central catheter occlusion in adult patients in the intensive care unit: a best practice implementation project. JBI Evid Implement. 2024;22:261–70. 38666477 10.1097/XEB.0000000000000426

[5] Fan CH Chu CN Chiu FH Chen C-T Tung H-H. Flushing and locking management related to central venous catheter occlusion rate among adult patients in acute care: a best practice implementation project. JBI Evid Implement. 2024;22:131–9. 37982206 10.1097/XEB.0000000000000394 PMC11107886

[6] Li HY Li YL Yu J. Evidence summary for prevention and management of central venous catheter occlusion. Chin J Nurs. 2022;57:2842–50.

[7] Santomauro I Campani D Tiozzo V. Heparin versus normal saline locking for prevention of occlusion, catheter-related infections and thrombosis in central venous catheter in adults: overview of systematic reviews. J Vasc Access. 2022;25:1741–8. 35686498 10.1177/11297298221103201

[8] Saunier J Khzam A Yagoubi N. Impact of mechanical stress on flexible tubing used for biomedical applications: characterization of the damages and impact on the patient’s health. J Mech Behav Biomed Mater. 2022;136:105477. 36219992 10.1016/j.jmbbm.2022.105477

[9] Okamura N Yamaoka I. A comparison of the effects of pulsatile and bolus flushing methods on lipid emulsion residues that lead to bacterial growth in intravenous catheters. J Vasc Access. 2024;25:1320–7. 37345317 10.1177/11297298231173162

[10] Gorski LA Hadaway L Hagle ME. Infusion therapy standards of practice, 8th edition. J Infus Nurs. 2021;44(suppl 1):S1–S224. 33394637 10.1097/NAN.0000000000000396

[11] Zhou M Dong S Zhang J. Effects of the low-speed continuous infusion catheter technique on double-lumen central venous catheters: a randomized controlled trial. Int J Nurs Stud. 2024;151:104676. 38241817 10.1016/j.ijnurstu.2023.104676

[12] Hosseini SJ Eidy F Kianmehr M. Comparing the effects of pulsatile and continuous flushing on time and type of peripheral intravenous catheters patency: a randomized clinical trial. J Caring Sci. 2021;10:84–8. 34222117 10.34172/jcs.2021.016 PMC8242293

[13] Hoff R Vervisch K De Coen K Smets K. Continuous infusion vs. intermittent flushing of peripheral cannulas in neonates using a needleless connector: a prospective cohort study. J Perinat Med. 2019;47:464–9. 30730844 10.1515/jpm-2018-0285

[14] Goossens GA De Waele Y Jerome M. Diagnostic accuracy of the Catheter Injection and Aspiration (CINAS) classification for assessing the function of totally implantable venous access devices. Supportive Care Cancer. 2016;24:755–61. 10.1007/s00520-015-2839-x 26209949

[15] Heath J Jones S. Utilization of an elastomeric continuous infusion device to maintain catheter patency. J Intraven Nurs. 2001;24:102–6. 11836834

[16] Rejane Rabelo-Silva E Lourenço SA Maestri RN. Patterns, appropriateness and outcomes of peripherally inserted central catheter use in Brazil: a multicentre study of 12 725 catheters. BMJ Qual Saf. 2022;31:652–61. 10.1136/bmjqs-2021-013869 PMC9411873 35086961

[17] Song X Lu H Chen F. A longitudinal observational retrospective study on risk factors and predictive model of PICC associated thrombosis in cancer patients. Sci Rep. 2020;10:10090. 32572092 10.1038/s41598-020-67038-x PMC7308336

[18] Zhang XF Li YL Yu J. Establishment and verification of the risk prediction model for central venous catheter occlusion. J Nurs Sci. 2020;35:35–8.

[19] Guiffant G Durussel JJ Merckx J Flaud P Vigier JP Mousset P. Flushing of intravascular access devices (IVADs) – efficacy of pulsed and continuous infusions. J Vasc Access. 2012;13:75–8. 21748725 10.5301/JVA.2011.8487

[20] Paquet F Marchionni C. What Is Your KVO? Historical perspectives, review of evidence, and a survey about an often overlooked nursing practice. J Infus Nurs. 2016;39:32–7. 26714117 10.1097/NAN.0000000000000147

