# Supplementary figures and images for: Use of continuous infusion technique with pre-filled elastic pumps for prevention of centrally inserted central catheter occlusion in critically ill patients: A feasibility study
Source: Medicine (Baltimore). 2024 Dec 13;103(50):e40930. doi: 10.1097/MD.0000000000040930 (PMC11651468; doi:10.1097/MD.0000000000040930)

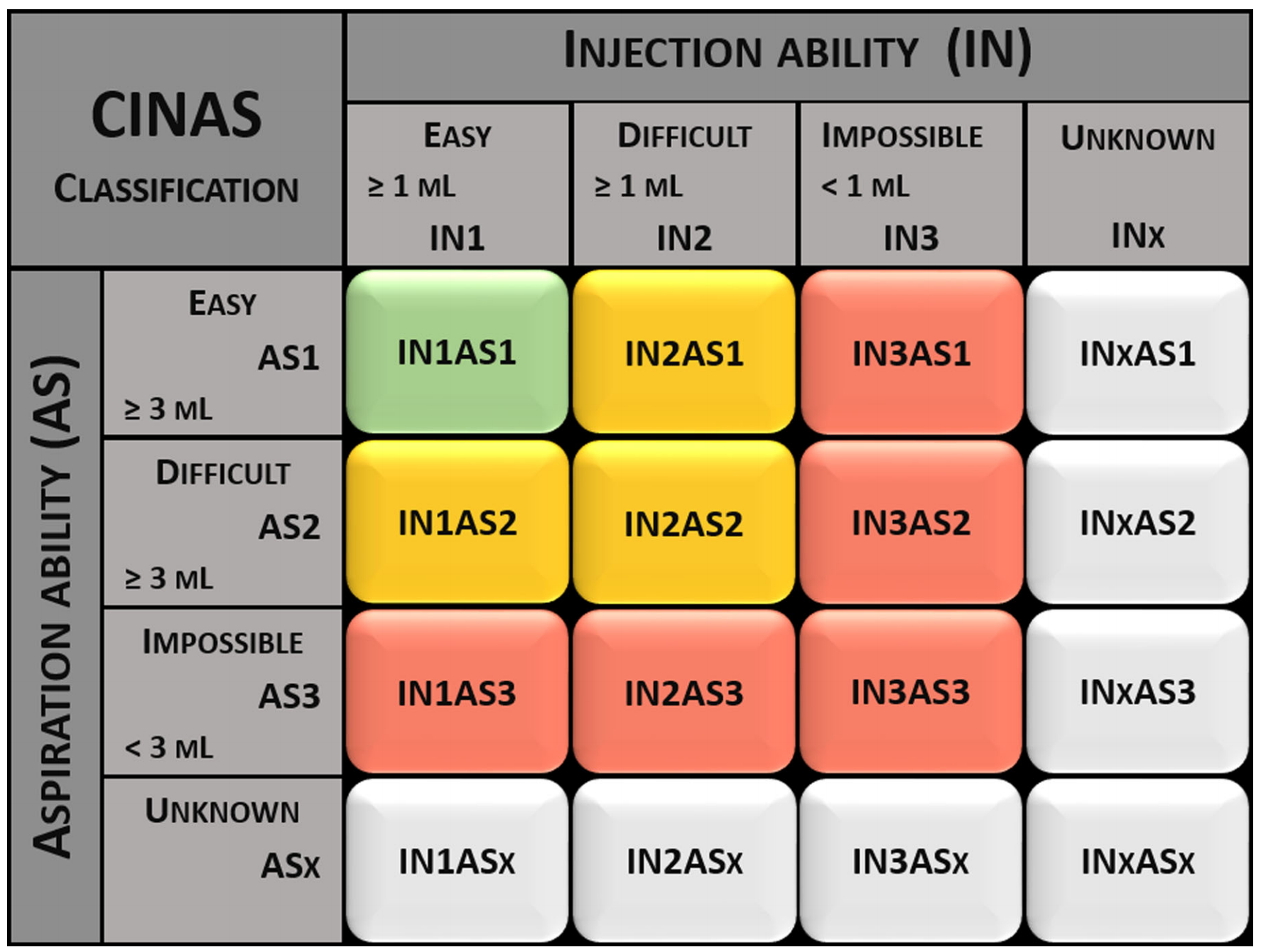

Supplement: Supplementary file 1 [file medi-103-e40930-s001.tif]
